# Statistical Conclusion Validity: Some Common Threats and Simple Remedies

**DOI:** 10.3389/fpsyg.2012.00325

**Published:** 2012-08-29

**Authors:** Miguel A. García-Pérez

**Affiliations:** ^1^Facultad de Psicología, Departamento de Metodología, Universidad ComplutenseMadrid, Spain

**Keywords:** data analysis, validity of research, regression, stopping rules, preliminary tests

## Abstract

The ultimate goal of research is to produce dependable knowledge or to provide the evidence that may guide practical decisions. Statistical conclusion validity (SCV) holds when the conclusions of a research study are founded on an adequate analysis of the data, generally meaning that adequate statistical methods are used whose small-sample behavior is accurate, besides being logically capable of providing an answer to the research question. Compared to the three other traditional aspects of research validity (external validity, internal validity, and construct validity), interest in SCV has recently grown on evidence that inadequate data analyses are sometimes carried out which yield conclusions that a proper analysis of the data would not have supported. This paper discusses evidence of three common threats to SCV that arise from widespread recommendations or practices in data analysis, namely, the use of repeated testing and optional stopping without control of Type-I error rates, the recommendation to check the assumptions of statistical tests, and the use of regression whenever a bivariate relation or the equivalence between two variables is studied. For each of these threats, examples are presented and alternative practices that safeguard SCV are discussed. Educational and editorial changes that may improve the SCV of published research are also discussed.

Psychologists are well aware of the traditional aspects of research validity introduced by Campbell and Stanley (1966) and further subdivided and discussed by Cook and Campbell (1979). Despite initial criticisms of the practically oriented and somewhat fuzzy distinctions among the various aspects (see Cook and Campbell, 1979, pp. 85–91; see also Shadish et al., 2002, pp. 462–484), the four facets of research validity have gained recognition and they are currently covered in many textbooks on research methods in psychology (e.g., Beins, 2009; Goodwin, 2010; Girden and Kabacoff, 2011). Methods and strategies aimed at securing research validity are also discussed in these and other sources. To simplify the description, *construct validity* is sought by using well-established definitions and measurement procedures for variables, *internal validity* is sought by ensuring that extraneous variables have been controlled and confounds have been eliminated, and *external validity* is sought by observing and measuring dependent variables under natural conditions or under an appropriate representation of them. The fourth aspect of research validity, which Cook and Campbell called *statistical conclusion validity* (SCV), is the subject of this paper.

Cook and Campbell, 1979, pp. 39–50) discussed that SCV pertains to the extent to which data from a research study can reasonably be regarded as revealing a link (or lack thereof) between independent and dependent variables *as far as statistical issues are concerned*. This particular facet was separated from other factors acting in the same direction (the three other facets of validity) and includes three aspects: (1) whether the study has enough statistical power to detect an effect if it exists, (2) whether there is a risk that the study will “reveal” an effect that does not actually exist, and (3) how can the magnitude of the effect be confidently estimated. They nevertheless considered the latter aspect as a mere step ahead once the first two aspects had been satisfactorily solved, and they summarized their position by stating that SCV “refers to inferences about whether it is reasonable to presume covariation given a specified α level and the obtained variances” (Cook and Campbell, 1979, p. 41). Given that mentioning “the obtained variances” was an indirect reference to statistical power and mentioning α was a direct reference to statistical significance, their position about SCV may have seemed to only entail consideration that the statistical decision can be incorrect as a result of Type-I and Type-II errors. Perhaps as a consequence of this literal interpretation, review papers studying SCV in published research have focused on power and significance (e.g., Ottenbacher, 1989; Ottenbacher and Maas, 1999), strategies aimed at increasing SCV have only considered these issues (e.g., Howard et al., 1983), and tutorials on the topic only or almost only mention these issues along with effect sizes (e.g., Orme, 1991; Austin et al., 1998; Rankupalli and Tandon, 2010). This emphasis on issues of significance and power may also be the reason that some sources refer to threats to SCV as “any factor that leads to a Type-I or a Type-II error” (e.g., Girden and Kabacoff, 2011, p. 6; see also Rankupalli and Tandon, 2010, Section 1.2), as if these errors had identifiable causes that could be prevented. It should be noted that SCV has also occasionally been purported to reflect the extent to which pre-experimental designs provide evidence for causation (Lee, 1985) or the extent to which meta-analyses are based on representative results that make the conclusion generalizable (Elvik, 1998).

But Cook and Campbell’s (1979, p. 80) aim was undoubtedly broader, as they stressed that SCV “is concerned with sources of random error *and with the appropriate use of statistics and statistical tests*” (italics added). Moreover, Type-I and Type-II errors are an essential and inescapable consequence of the statistical decision theory underlying significance testing and, as such, the potential occurrence of one or the other of these errors cannot be prevented. The actual occurrence of them for the data on hand cannot be assessed either. Type-I and Type-II errors will always be with us and, hence, SCV is only trivially linked to the fact that research will never unequivocally prove or reject any statistical null hypothesis or its originating research hypothesis. Cook and Campbell seemed to be well aware of this issue when they stressed that SCV refers to reasonable inferences given a specified significance level and a given power. In addition, Stevens (1950, p. 121) forcefully emphasized that “*it is a statistician’s duty to be wrong* the stated number of times,” implying that a researcher should accept the assumed risks of Type-I and Type-II errors, use statistical methods that guarantee the assumed error rates, and consider these as an essential part of the research process. From this position, these errors do not affect SCV unless their probability differs meaningfully from that which was assumed. And this is where an alternative perspective on SCV enters the stage, namely, whether the data were analyzed *properly* so as to extract conclusions that faithfully reflect what the data have to say about the research question. A negative answer raises concerns about SCV beyond the triviality of Type-I or Type-II errors. There are actually two types of threat to SCV from this perspective. One is when the data are subjected to thoroughly inadequate statistical analyses that do not match the characteristics of the design used to collect the data or that cannot logically give an answer to the research question. The other is when a proper statistical test is used but it is applied under conditions that alter the stated risk probabilities. In the former case, the conclusion will be wrong except by accident; in the latter, the conclusion will fail to be incorrect with the declared probabilities of Type-I and Type-II errors.

The position elaborated in the foregoing paragraph is well summarized in Milligan and McFillen’s (1984, p. 439) statement that “under normal conditions (…) the researcher will not know when a null effect has been declared significant or when a valid effect has gone undetected (…) Unfortunately, the statistical conclusion validity, and the ultimate value of the research, rests on the explicit control of (Type-I and Type-II) error rates.” This perspective on SCV is explicitly discussed in some textbooks on research methods (e.g., Beins, 2009, pp. 139–140; Goodwin, 2010, pp. 184–185) and some literature reviews have been published that reveal a sound failure of SCV in these respects.

For instance, Milligan and McFillen’s (1984, p. 438) reviewed evidence that “the business research community has succeeded in publishing a great deal of incorrect and statistically inadequate research” and they dissected and discussed in detail four additional cases (among many others that reportedly could have been chosen) in which a breach of SCV resulted from gross mismatches between the research design and the statistical analysis. Similarly, García-Pérez (2005) reviewed alternative methods to compute confidence intervals for proportions and discussed three papers (among many others that reportedly could have been chosen) in which inadequate confidence intervals had been computed. More recently, Bakker and Wicherts (2011) conducted a thorough analysis of psychological papers and estimated that roughly 50% of published papers contain reporting errors, although they only checked whether the reported *p* value was correct and not whether the statistical test used was appropriate. A similar analysis carried out by Nieuwenhuis et al. (2011) revealed that 50% of the papers reporting the results of a comparison of two experimental effects in top neuroscience journals had used an incorrect statistical procedure. And Bland and Altman (2011) reported further data on the prevalence of incorrect statistical analyses of a similar nature.

An additional indicator of the use of inadequate statistical procedures arises from consideration of published papers whose title explicitly refers to a re-analysis of data reported in some other paper. A literature search for papers including in their title the terms “a re-analysis,” “a reanalysis,” “re-analyses,” “reanalyses,” or “alternative analysis” was conducted on May 3, 2012 in the Web of Science (WoS; http://thomsonreuters.com), which rendered 99 such papers with subject area “Psychology” published in 1990 or later. Although some of these were false positives, a sizeable number of them actually discussed the inadequacy of analyses carried out by the original authors and reported the results of proper alternative analyses that typically reversed the original conclusion. This type of outcome upon re-analyses of data are more frequent than the results of this quick and simple search suggest, because the information for identification is not always included in the title of the paper or is included in some other form: For a simple example, the search for the clause “a closer look” in the title rendered 131 papers, many of which also presented re-analyses of data that reversed the conclusion of the original study.

Poor design or poor sample size planning may, unbeknownst to the researcher, lead to unacceptable Type-II error rates, which will certainly affect SCV (as long as the null is not rejected; if it is, the probability of a Type-II error is irrelevant). Although insufficient power due to lack of proper planning has consequences on statistical tests, the thread of this paper de-emphasizes this aspect of SCV (which should perhaps more reasonably fit within an alternative category labeled *design validity*) and emphasizes the idea that SCV holds when statistical conclusions are incorrect with the stated probabilities of Type-I and Type-II errors (whether the latter was planned or simply computed). Whether or not the actual significance level used in the research or the power that it had is judged acceptable is another issue, which does not affect SCV: The statistical conclusion is valid within the stated (or computed) error probabilities. A breach of SCV occurs, then, when the data are not subjected to adequate statistical analyses or when control of Type-I or Type-II errors is lost.

It should be noted that a further component was included into consideration of SCV in Shadish et al.’s (2002) sequel to Cook and Campbell’s (1979) book, namely, effect size. Effect size relates to what has been called a Type-III error (Crawford et al., 1998), that is, a statistically significant result that has no meaningful practical implication and that only arises from the use of a huge sample. This issue is left aside in the present paper because adequate consideration and reporting of effect sizes precludes Type-III errors, although the recommendations of Wilkinson and The Task Force on Statistical Inference (1999) in this respect are not always followed. Consider, e.g., Lippa’s (2007) study of the relation between sex drive and sexual attraction. Correlations generally lower than 0.3 in absolute value were declared strong as a result of *p* values below 0.001. With sample sizes sometimes nearing 50,000 paired observations, even correlations valued at 0.04 turned out significant in this study. More attention to effect sizes is certainly needed, both by researchers and by journal editors and reviewers.

The remainder of this paper analyzes three common practices that result in SCV breaches, also discussing simple replacements for them.

## Stopping Rules for Data Collection without Control of Type-I Error Rates

The asymptotic theory that provides justification for null hypothesis significance testing (NHST) assumes what is known as *fixed sampling*, which means that the size *n* of the sample is not itself a random variable or, in other words, that the size of the sample has been decided in advance and the statistical test is performed once the entire sample of data has been collected. Numerous procedures have been devised to determine the size that a sample must have according to planned power (Ahn et al., 2001; Faul et al., 2007; Nisen and Schwertman, 2008; Jan and Shieh, 2011), the size of the effect sought to be detected (Morse, 1999), or the width of the confidence intervals of interest (Graybill, 1958; Boos and Hughes-Oliver, 2000; Shieh and Jan, 2012). For reviews, see Dell et al. (2002) and Maxwell et al. (2008). In many cases, a researcher simply strives to gather as large a sample as possible. Asymptotic theory supports NHST under fixed sampling assumptions, whether or not the size of the sample was planned.

In contrast to fixed sampling, *sequential sampling* implies that the number of observations is not fixed in advance but depends by some rule on the observations already collected (Wald, 1947; Anscombe, 1953; Wetherill, 1966). In practice, data are analyzed as they come in and data collection stops when the observations collected thus far satisfy some criterion. The use of sequential sampling faces two problems (Anscombe, 1953, p. 6): (i) devising a suitable stopping rule and (ii) finding a suitable test statistic and determining its sampling distribution. The mere statement of the second problem evidences that the sampling distribution of conventional test statistics for fixed sampling no longer holds under sequential sampling. These sampling distributions are relatively easy to derive in some cases, particularly in those involving negative binomial parameters (Anscombe, 1953; García-Pérez and Núñez-Antón, 2009). The choice between fixed and sequential sampling (sometimes portrayed as the “experimenter’s intention”; see Wagenmakers, 2007) has important ramifications for NHST because the probability that the observed data are compatible (by any criterion) with a true null hypothesis generally differs greatly across sampling methods. This issue is usually bypassed by those who look at the data as a “sure fact” once collected, as if the sampling method used to collect the data did not make any difference or should not affect how the data are interpreted.

There are good reasons for using sequential sampling in psychological research. For instance, in clinical studies in which patients are recruited on the go, the experimenter may want to analyze data as they come in to be able to prevent the administration of a seemingly ineffective or even hurtful treatment to new patients. In studies involving a waiting-list control group, individuals in this group are generally transferred to an experimental group midway along the experiment. In studies with laboratory animals, the experimenter may want to stop testing animals before the planned number has been reached so that animals are not wasted when an effect (or the lack thereof) seems established. In these and analogous cases, the decision as to whether data will continue to be collected results from an analysis of the data collected thus far, typically using a statistical test that was devised for use in conditions of fixed sampling. In other cases, experimenters test their statistical hypothesis each time a new observation or block of observations is collected, and continue the experiment until they feel the data are conclusive one way or the other. Software has been developed that allows experimenters to find out how many more observations will be needed for a marginally non-significant result to become significant on the assumption that sample statistics will remain invariant when the extra data are collected (Morse, 1998).

The practice of repeated testing and optional stopping has been shown to affect in unpredictable ways the empirical Type-I error rate of statistical tests designed for use under fixed sampling (Anscombe, 1954; Armitage et al., 1969; McCarroll et al., 1992; Strube, 2006; Fitts, 2011a). The same holds when a decision is made to collect further data on evidence of a marginally (non) significant result (Shun et al., 2001; Chen et al., 2004). The inaccuracy of statistical tests in these conditions represents a breach of SCV, because the statistical conclusion thus fails to be incorrect with the assumed (and explicitly stated) probabilities of Type-I and Type-II errors. But there is an easy way around the inflation of Type-I error rates from within NHST, which solves the threat to SCV that repeated testing and optional stopping entail.

In what appears to be the first development of a sequential procedure with control of Type-I error rates in psychology, Frick (1998) proposed that repeated statistical testing be conducted under the so-called COAST (composite open adaptive sequential test) rule: If the test yields *p* < 0.01, stop collecting data and reject the null; if it yields *p* > 0.36, stop also and do not reject the null; otherwise, collect more data and re-test. The *low criterion* at 0.01 and the *high criterion* at 0.36 were selected through simulations so as to ensure a final Type-I error rate of 0.05 for paired-samples *t* tests. Use of the same low and high criteria rendered similar control of Type-I error rates for tests of the product-moment correlation, but they yielded slightly conservative tests of the interaction in 2 × 2 between-subjects ANOVAs. Frick also acknowledged that adjusting the low and high criteria might be needed in other cases, although he did not address them. This has nevertheless been done by others who have modified and extended Frick’s approach (e.g., Botella et al., 2006; Ximenez and Revuelta, 2007; Fitts, 2010a,b, 2011b). The result is sequential procedures with stopping rules that guarantee accurate control of final Type-I error rates for the statistical tests that are more widely used in psychological research.

Yet, these methods do not seem to have ever been used in actual research, or at least their use has not been acknowledged. For instance, of the nine citations to Frick’s (1998) paper listed in WoS as of May 3, 2012, only one is from a paper (published in 2011) in which the COAST rule was reportedly used, although unintendedly. And not a single citation is to be found in WoS from papers reporting the use of the extensions and modifications of Botella et al. (2006) or Ximenez and Revuelta (2007). Perhaps researchers in psychology invariably use fixed sampling, but it is hard to believe that “data peeking” or “data monitoring” was never used, or that the results of such interim analyses never led researchers to collect some more data. Wagenmakers (2007, p. 785) regretted that “it is not clear what percentage of *p* values reported in experimental psychology have been contaminated by some form of optional stopping. There is simply no information in Results sections that allows one to assess the extent to which optional stopping has occurred.” This incertitude was quickly resolved by John et al. (2012). They surveyed over 2000 psychologists with highly revealing results: Respondents affirmatively admitted to the practices of data peeking, data monitoring, or conditional stopping in rates that varied between 20 and 60%.

Besides John et al.’s (2012) proposal that authors disclose these details in full and Simmons et al.’s (2011) proposed list of requirements for authors and guidelines for reviewers, the solution to the problem is simple: Use strategies that control Type-I error rates upon repeated testing and optional stopping. These strategies have been widely used in biomedical research for decades (Bauer and Köhne, 1994; Mehta and Pocock, 2011). There is no reason that psychological research should ignore them and give up efficient research with control of Type-I error rates, particularly when these strategies have also been adapted and further developed for use under the most common designs in psychological research (Frick, 1998; Botella et al., 2006; Ximenez and Revuelta, 2007; Fitts, 2010a,b).

It should also be stressed that not all instances of repeated testing or optional stopping without control of Type-I error rates threaten SCV. A breach of SCV occurs only when the conclusion regarding the research question is based on the use of these practices. For an acceptable use, consider the study of Xu et al. (2011). They investigated order preferences in primates to find out whether primates preferred to receive the best item first rather than last. Their procedure involved several experiments and they declared that “three significant sessions (two-tailed binomial tests per session, *p* < 0.05) or 10 consecutive non-significant sessions were required from each monkey before moving to the next experiment. The three significant sessions were not necessarily consecutive (…) Ten consecutive non-significant sessions were taken to mean there was no preference by the monkey” (p. 2304). In this case, the use of repeated testing with optional stopping at a nominal 95% significance level for each individual test is part of the operational definition of an outcome variable used as a criterion to proceed to the next experiment. And, in any event, the overall probability of misclassifying a monkey according to this criterion is certainly fixed at a known value that can easily be worked out from the significance level declared for each individual binomial test. One may object to the value of the resultant risk of misclassification, but this does not raise concerns about SCV.

In sum, the use of repeated testing with optional stopping threatens SCV for lack of control of Type-I and Type-II error rates. A simple way around this is to refrain from these practices and adhere to the fixed sampling assumptions of statistical tests; otherwise, use the statistical methods that have been developed for use with repeated testing and optional stopping.

## Preliminary Tests of Assumptions

To derive the sampling distribution of test statistics used in parametric NHST, some assumptions must be made about the probability distribution of the observations or about the parameters of these distributions. The assumptions of normality of distributions (in all tests), homogeneity of variances (in Student’s two-sample *t* test for means or in ANOVAs involving between-subjects factors), sphericity (in repeated-measures ANOVAs), homoscedasticity (in regression analyses), or homogeneity of regression slopes (in ANCOVAs) are well known cases. The data on hand may or may not meet these assumptions and some parametric tests have been devised under alternative assumptions (e.g., Welch’s test for two-sample means, or correction factors for the degrees of freedom of *F* statistics from ANOVAs). Most introductory statistics textbooks emphasize that the assumptions underlying statistical tests must be formally tested to guide the choice of a suitable test statistic for the null hypothesis of interest. Although this recommendation seems reasonable, serious consequences on SCV arise from following it.

Numerous studies conducted over the past decades have shown that the two-stage approach of testing assumptions first and subsequently testing the null hypothesis of interest has severe effects on Type-I and Type-II error rates. It may seem at first sight that this is simply the result of cascaded binary decisions each of which has its own Type-I and Type-II error probabilities; yet, this is the result of more complex interactions of Type-I and Type-II error rates that do not have fixed (empirical) probabilities across the cases that end up treated one way or the other according to the outcomes of the preliminary test: The resultant Type-I and Type-II error rates of the conditional test cannot be predicted from those of the preliminary and conditioned tests. A thorough analysis of what factors affect the Type-I and Type-II error rates of two-stage approaches is beyond the scope of this paper but readers should be aware that nothing suggests in principle that a two-stage approach might be adequate. The situations that have been more thoroughly studied include preliminary goodness-of-fit tests for normality before conducting a one-sample *t* test (Easterling and Anderson, 1978; Schucany and Ng, 2006; Rochon and Kieser, 2011), preliminary tests of equality of variances before conducting a two-sample *t* test for means (Gans, 1981; Moser and Stevens, 1992; Zimmerman, 1996, 2004; Hayes and Cai, 2007), preliminary tests of both equality of variances and normality preceding two-sample *t* tests for means (Rasch et al., 2011), or preliminary tests of homoscedasticity before regression analyses (Caudill, 1988; Ng and Wilcox, 2011). These and other studies provide evidence that strongly advises against conducting preliminary tests of assumptions. Almost all of these authors explicitly recommended against these practices and hoped for the misleading and misguided advice given in introductory textbooks to be removed. Wells and Hintze (2007, p. 501) concluded that “checking the assumptions using the same data that are to be analyzed, although attractive due to its empirical nature, is a fruitless endeavor because of its negative ramifications on the actual test of interest.” The ramifications consist of substantial but unknown alterations of Type-I and Type-II error rates and, hence, a breach of SCV.

Some authors suggest that the problem can be solved by replacing the formal test of assumptions with a decision based on a suitable graphical display of the data that helps researchers judge by eye whether the assumption is tenable. It should be emphasized that the problem still remains, because the decision on how to analyze the data is conditioned on the results of a preliminary analysis. The problem is not brought about by a formal preliminary test, but by the conditional approach to data analysis. The use of a non-formal preliminary test only prevents a precise investigation of the consequences on Type-I and Type-II error rates. But the “out of sight, out of mind” philosophy does not eliminate the problem.

It thus seems that a researcher must make a choice between two evils: either not testing assumptions (and, thus, threatening SCV as a result of the uncontrolled Type-I and Type-II error rates that arise from a potentially undue application of the statistical test) or testing them (and, then, also losing control of Type-I and Type-II error rates owing to the two-stage approach). Both approaches are inadequate, as applying non-robust statistical tests to data that do not satisfy the assumptions has generally as severe implications on SCV as testing preliminary assumptions in a two-stage approach. One of the solutions to the dilemma consists of switching to statistical procedures that have been designed for use under the two-stage approach. For instance, Albers et al. (2000) used second-order asymptotics to derive the size and power of a two-stage test for independent means preceded by a test of equality of variances. Unfortunately, derivations of this type are hard to carry out and, hence, they are not available for most of the cases of interest. A second solution consists of using classical test statistics that have been shown to be robust to violation of their assumptions. Indeed, dependable unconditional tests for means or for regression parameters have been identified (see Sullivan and D’Agostino, 1992; Lumley et al., 2002; Zimmerman, 2004, 2011; Hayes and Cai, 2007; Ng and Wilcox, 2011). And a third solution is switching to modern robust methods (see, e.g., Wilcox and Keselman, 2003; Keselman et al., 2004; Wilcox, 2006; Erceg-Hurn and Mirosevich, 2008; Fried and Dehling, 2011).

Avoidance of the two-stage approach in either of these ways will restore SCV while observing the important requirement that statistical methods should be used whose assumptions are not violated by the characteristics of the data.

## Regression as a Means to Investigate Bivariate Relations of all Types

Correlational methods define one of the branches of scientific psychology (Cronbach, 1957) and they are still widely used these days in some areas of psychology. Whether in regression analyses or in latent variable analyses (Bollen, 2002), vast amounts of data are subjected to these methods. Regression analyses rely on an assumption that is often overlooked in psychology, namely, that the predictor variables have fixed values and are measured without error. This assumption, whose validity can obviously be assessed without recourse to any preliminary statistical test, is listed in all statistics textbooks.

In some areas of psychology, predictors actually have this characteristic because they are physical variables defining the magnitude of stimuli, and any error with which these magnitudes are measured (or with which stimuli with the selected magnitudes are created) is negligible in practice. Among others, this is the case in psychophysical studies aimed at estimating *psychophysical functions* describing the form of the relation between physical magnitude and perceived magnitude (e.g., Green, 1982) or *psychometric functions* describing the form of the relation between physical magnitude and performance in a detection, discrimination, or identification task (Armstrong and Marks, 1997; Saberi and Petrosyan, 2004; García-Pérez et al., 2011). Regression or analogous methods are typically used to estimate the parameters of these relations, with stimulus magnitude as the independent variable and perceived magnitude (or performance) as the dependent variable. The use of regression in these cases is appropriate because the independent variable has fixed values measured without error (or with a negligible error). Another area in which the use of regression is permissible is in simulation studies on parameter recovery (García-Pérez et al., 2010), where the true parameters generating the data are free of measurement error by definition.

But very few other predictor variables used in psychology meet this requirement, as they are often test scores or performance measures that are typically affected by non-negligible and sometimes large measurement error. This is the case of the proportion of hits and the proportion of false alarms in psychophysical tasks, whose theoretical relation is linear under some signal detection models (DeCarlo, 1998) and, thus, suggests the use of simple linear regression to estimate its parameters. Simple linear regression is also sometimes used as a complement to statistical tests of equality of means in studies in which equivalence or agreement is assessed (e.g., Maylor and Rabbitt, 1993; Baddeley and Wilson, 2002), and in these cases equivalence implies that the slope should not differ significantly from unity and that the intercept should not differ significantly from zero. The use of simple linear regression is also widespread in priming studies after Greenwald et al. (1995; see also Draine and Greenwald, 1998), where the intercept (and sometimes the slope) of the linear regression of priming effect on detectability of the prime are routinely subjected to NHST.

In all the cases just discussed and in many others where the *X* variable in the regression of *Y* on *X* is measured with error, a study of the relation between *X* and *Y* through regression is inadequate and has serious consequences on SCV. The least of these problems is that there is no basis for assigning the roles of independent and dependent variable in the regression equation (as a non-directional relation exists between the variables, often without even a temporal precedence relation), but regression parameters will differ according to how these roles are assigned. In influential papers of which most researchers in psychology seem to be unaware, Wald (1940) and Mandansky (1959) distinguished regression relations from structural relations, the latter reflecting the case in which both variables are measured with error. Both authors illustrated the consequences of fitting a regression line when a structural relation is involved and derived suitable estimators and significance tests for the slope and intercept parameters of a structural relation. This topic was brought to the attention of psychologists by Isaac (1970) in a criticism of Treisman and Watts’ (1966) use of simple linear regression to assess the equivalence of two alternative estimates of psychophysical sensitivity (*d*′ measures from signal detection theory analyses). The difference between regression and structural relations is briefly mentioned in passing in many elementary books on regression, the issue of fitting structural relations (sometimes referred to as *Deming’s regression* or the *errors-in-variables regression model*) is addressed in detail in most intermediate and advance books on regression (e.g., Fuller, 1987; Draper and Smith, 1998) and hands-on tutorials have been published (e.g., Cheng and Van Ness, 1994; Dunn and Roberts, 1999; Dunn, 2007). But this type of analysis is not in the toolbox of the average researcher in psychology^1^. In contrast, recourse to this type analysis is quite common in the biomedical sciences.

Use of this commendable method may generalize when researchers realize that estimates of the slope β and the intercept α of a structural relation can be easily computed through

$$\begin{array}{rcll} \hat{\beta } & =\frac{S_{y}^{2}-\lambda S_{x}^{2}+\sqrt{{\left(S_{y}^{2}-\lambda S_{x}^{2}\right)}^{2}+4\lambda S_{xy}^{2}}}{2S_{xy}}, & & \text{(1)}\\ \hat{\alpha } & =Ȳ-\hat{\beta }\bar{X}, & & \text{(2)} \end{array}$$

where $\bar{X},$ Ȳ, $S_{x}^{2},$ $S_{y}^{2},$ and $S_{xy}$ are the sample means, variances, and covariance of *X* and *Y*, and $\lambda =\sigma_{\varepsilon_{y}}^{2}∕\sigma_{\varepsilon_{x}}^{2}$ is the ratio of the variances of measurement errors in *Y* and in *X*. When *X* and *Y* are the same variable measured at different times or under different conditions (as in Maylor and Rabbitt, 1993; Baddeley and Wilson, 2002), λ = 1 can safely be assumed (for an actual application, see Smith et al., 2004). In other cases, a rough estimate can be used, as the estimates of α and β have been shown to be robust except under extreme departures of the guesstimated λ from its true value (Ketellapper, 1983).

**Figure 1:**
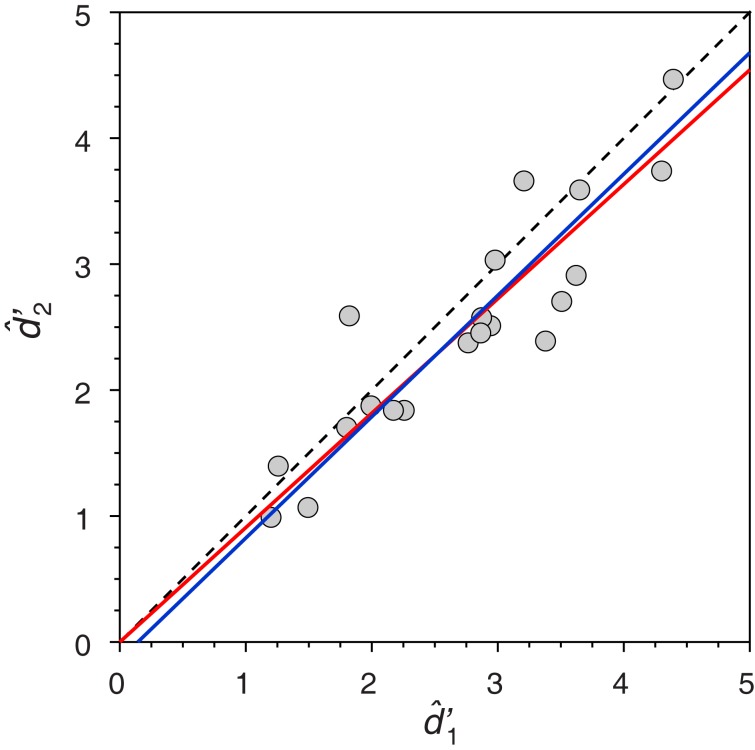
**Replot of data from Yeshurun et al. (2008, their Figure 8) with their fitted regression line through the origin (red line) and a fitted structural relation (blue line)**. The identity line is shown with dashed trace for comparison. For additional analyses bearing on the SCV of the original study, see García-Pérez and Alcalá-Quintana (2011).

For illustration, consider Yeshurun et al. (2008) comparison of signal detection theory estimates of *d*′ in each of the intervals of a two alternative forced-choice task, which they pronounced different as revealed by a regression analysis through the origin. Note that this is the context in which Isaac (1970) had illustrated the inappropriateness of regression. The data are shown in Figure 1, and Yeshurun et al. rejected equality of $d_{1}^{\prime }$ and $d_{2}^{\prime }$ because the regression slope through the origin (red line, whose slope is 0.908) differed significantly from unity: The 95% confidence interval for the slope ranged between 0.844 and 0.973. Using Eqs 1 and 2, the estimated structural relation is instead given by the blue line in Figure 1. The difference seems minor by eye, but the slope of the structural relation is 0.963, which is not significantly different from unity (*p* = 0.738, two-tailed; see Isaac, 1970, p. 215). This outcome, which reverses a conclusion raised upon inadequate data analyses, is representative of other cases in which the null hypothesis *H*_0_: β = 1 was rejected. The reason is dual: (1) the slope of a structural relation is estimated with severe bias through regression (Riggs et al., 1978; Kalantar et al., 1995; Hawkins, 2002) and (2) regression-based statistical tests of *H*_0_: β = 1 render empirical Type-I error rates that are much higher than the nominal rate when both variables are measured with error (García-Pérez and Alcalá-Quintana, 2011).

In sum, SCV will improve if structural relations instead of regression equations were fitted when both variables are measured with error.

## Conclusion

Type-I and Type-II errors are essential components of the statistical decision theory underlying NHST and, therefore, data can never be expected to answer a research question unequivocally. This paper has promoted a view of SCV that de-emphasizes consideration of these unavoidable errors and considers instead two alternative issues: (1) whether statistical tests are used that match the research design, goals of the study, and formal characteristics of the data and (2) whether they are applied in conditions under which the resultant Type-I and Type-II error rates match those that are declared as limiting the validity of the conclusion. Some examples of common threats to SCV in these respects have been discussed and simple and feasible solutions have been proposed. For reasons of space, another threat to SCV has not been covered in this paper, namely, the problems arising from multiple testing (i.e., in concurrent tests of more than one hypothesis). Multiple testing is commonplace in brain mapping studies and some implications on SCV have been discussed, e.g., by Bennett et al. (2009), Vul et al. (2009a,b), and Vecchiato et al. (2010).

All the discussion in this paper has assumed the frequentist approach to data analysis. In closing, and before commenting on how SCV could be improved, a few words are worth about how Bayesian approaches fare on SCV.

### The Bayesian approach

Advocates of Bayesian approaches to data analysis, hypothesis testing, and model selection (e.g., Jennison and Turnbull, 1990; Wagenmakers, 2007; Matthews, 2011) overemphasize the problems of the frequentist approach and praise the solutions offered by the Bayesian approach: Bayes factors (BFs) for hypothesis testing, credible intervals for interval estimation, Bayesian posterior probabilities, Bayesian information criterion (BIC) as a tool for model selection and, above all else, strict reliance on observed data and independence of the sampling plan (i.e., fixed vs. sequential sampling). There is unquestionable merit in these alternatives and a fair comparison with their frequentist counterparts requires a detailed analysis that is beyond the scope of this paper. Yet, I cannot resist the temptation of commenting on the presumed problems of the frequentist approach and also on the standing of the Bayesian approach with respect to SCV.

One of the preferred objections to *p* values is that they relate to data that were never collected and which, thus, should not affect the decision of what hypothesis the observed data support or fail to support. Intuitively appealing as it may seem, the argument is flawed because the referent for a *p* value is not other data sets that could have been observed in undone replications of the same experiment. Instead, the referent is the properties of the test statistic itself, which is guaranteed to have the declared sampling distribution when data are collected as assumed in the derivation of such distribution. Statistical tests are calibrated procedures with known properties, and this calibration is what makes their results interpretable. As is the case for any other calibrated procedure or measuring instrument, the validity of the outcome only rests on adherence to the usage specifications. And, of course, the test statistic and the resultant *p* value on application cannot be blamed for the consequences of a failure to collect data properly or to apply the appropriate statistical test.

Consider a two-sample *t* test for means. Those who need a referent may want to notice that the *p* value for the data from a given experiment relates to the uncountable times that such test has been applied to data from any experiment in any discipline. Calibration of the *t* test ensures that a proper use with a significance level of, say, 5% will reject a true null hypothesis on 5% of the occasions, no matter what the experimental hypothesis is, what the variables are, what the data are, what the experiment is about, who carries it out, or in what research field. What a *p* value indicates is how tenable it is that the *t* statistic will attain the observed value if the null were correct, with only a trivial link to the data observed in the experiment of concern. And this only places in a precise quantitative framework the logic that the man on the street uses to judge, for instance, that getting struck by lightning four times over the past 10 years is not something that could identically have happened to anybody else, or that the source of a politician’s huge and untraceable earnings is not the result of allegedly winning top lottery prizes numerous times over the past couple of years. In any case, the advantage of the frequentist approach as regards SCV is that the probability of a Type-I or a Type-II error can be clearly and unequivocally stated, which is not to be mistaken for a statement that a *p* value is the probability of a Type-I error in the current case, or that it is a measure of the strength of evidence against the null that the current data provide. The most prevalent problems of *p* values are their potential for misuse and their widespread misinterpretation (Nickerson, 2000). But misuse or misinterpretation do not make NHST and *p* values uninterpretable or worthless.

Bayesian approaches are claimed to be free of these presumed problems, yielding a conclusion that is exclusively grounded on the data. In a naive account of Bayesian hypothesis testing, Malakoff (1999) attributes to biostatistician Steven Goodman the assertion that the Bayesian approach “says there is an X% probability that your hypothesis is true–not that there is some convoluted chance that if you assume the null hypothesis is true, you will get a similar or more extreme result if you repeated your experiment thousands of times.” Besides being misleading and reflecting a poor understanding of the logic of calibrated NHST methods, what goes unmentioned in this and other accounts is that the Bayesian potential to find out the probability that the hypothesis is true will not materialize without two crucial extra pieces of information. One is the *a priori* probability of each of the competing hypotheses, which certainly does not come from the data. The other is the probability of the observed data under each of the competing hypothesis, which has the same origin as the frequentist *p* value and whose computation requires distributional assumptions that must necessarily take the sampling method into consideration.

In practice, Bayesian hypothesis testing generally computes BFs and the result might be stated as “the alternative hypothesis is *x* times more likely than the null,” although the probability that this type of statement is wrong is essentially unknown. The researcher may be content with a conclusion of this type, but how much of these odds comes from the data and how much comes from the extra assumptions needed to compute a BF is undecipherable. In many cases research aims at gathering and analyzing data to make informed decisions such as whether application of a treatment should be discontinued, whether changes should be introduced in an educational program, whether daytime headlights should be enforced, or whether in-car use of cell phones should be forbidden. Like frequentist analyses, Bayesian approaches do not guarantee that the decisions will be correct. One may argue that stating how much more likely is one hypothesis over another bypasses the decision to reject or not reject any of them and, then, that Bayesian approaches to hypothesis testing are free of Type-I and Type-II errors. Although this is technically correct, the problem remains from the perspective of SCV: Statistics is only a small part of a research process whose ultimate goal is to reach a conclusion and make a decision, and researchers are in a better position to defend their claims if they can supplement them with a statement of the probability with which those claims are wrong.

Interestingly, analyses of decisions based on Bayesian approaches have revealed that they are no better than frequentist decisions as regards Type-I and Type-II errors and that parametric assumptions (i.e., the choice of prior and the assumed distribution of the observations) crucially determine the performance of Bayesian methods. For instance, Bayesian estimation is also subject to potentially large bias and lack of precision (Alcalá-Quintana and García-Pérez, 2004; García-Pérez and Alcalá-Quintana, 2007), the coverage probability of Bayesian credible intervals can be worse than that of frequentist confidence intervals (Agresti and Min, 2005; Alcalá-Quintana and García-Pérez, 2005), and the Bayesian posterior probability in hypothesis testing can be arbitrarily large or small (Zaslavsky, 2010). On another front, use of BIC for model selection may discard a true model as often as 20% of the times, while a concurrent 0.05-size chi-square test rejects the true model between 3 and 7% of times, closely approximating its stated performance (García-Pérez and Alcalá-Quintana, 2012). In any case, the probabilities of Type-I and Type-II errors in practical decisions made from the results of Bayesian analyses will always be unknown and beyond control.

### Improving the SCV of research

Most breaches of SCV arise from a poor understanding of statistical procedures and the resultant inadequate usage. These problems can be easily corrected, as illustrated in this paper, but the problems will not have arisen if researchers had had a better statistical training in the first place. There was a time in which one simply could not run statistical tests without a moderate understanding of NHST. But these days the application of statistical tests is only a mouse-click away and all that students regard as necessary is learning the rule by which *p* values pouring out of statistical software tell them whether the hypothesis is to be accepted or rejected, as the study of Hoekstra et al. (2012) seems to reveal.

One way to eradicate the problem is by improving statistical education at undergraduate and graduate levels, perhaps not just focusing on giving formal training on a number of methods but by providing students with the necessary foundations that will subsequently allow them to understand and apply methods for which they received no explicit formal training. In their analysis of statistical errors in published papers, Milligan and McFillen (1984, p. 461) concluded that “in doing projects, it is not unusual for applied researchers or students to use or apply a statistical procedure for which they have received no formal training. This is as inappropriate as a person conducting research in a given content area before reading the existing background literature on the topic. The individual simply is not prepared to conduct quality research. The attitude that statistical technology is secondary or less important to a person’s formal training is shortsighted. Researchers are unlikely to master additional statistical concepts and techniques after leaving school. Thus, the statistical training in many programs must be strengthened. A single course in experimental design and a single course in multivariate analysis is probably insufficient for the typical student to master the course material. Someone who is trained only in theory and content will be ill-prepared to contribute to the advancement of the field or to critically evaluate the research of others.” But statistical education does not seem to have changed much over the subsequent 25 years, as revealed by survey studies conducted by Aiken et al. (1990), Friedrich et al. (2000), Aiken et al. (2008), and Henson et al. (2010). Certainly some work remains to be done in this arena, and I can only second the proposals made in the papers just cited. But there is also the problem of the unhealthy over-reliance on narrow-breadth, clickable software for data analysis, which practically obliterates any efforts that are made to teach and promote alternatives (see the list of “Pragmatic Factors” discussed by Borsboom, 2006, pp. 431–434).

The last trench in the battle against breaches of SCV is occupied by journal editors and reviewers. Ideally, they also watch for problems in these respects. There is no known in-depth analysis of the review process in psychology journals (but see Nickerson, 2005) and some evidence reveals that the focus of the review process is not always on the quality or validity of the research (Sternberg, 2002; Nickerson, 2005). Simmons et al. (2011) and Wicherts et al. (2012) have discussed empirical evidence of inadequate research and review practices (some of which threaten SCV) and they have proposed detailed schemes through which feasible changes in editorial policies may help eradicate not only common threats to SCV but also other threats to research validity in general. I can only second proposals of this type. Reviewers and editors have the responsibility of filtering out (or requesting amendments to) research that does not meet the journal’s standards, including SCV. The analyses of Milligan and McFillen (1984) and Nieuwenhuis et al. (2011) reveal a sizeable number of published papers with statistical errors. This indicates that some remains to be done in this arena too, and some journals have indeed started to take action (see Aickin, 2011).

## Conflict of Interest Statement

The author declares that the research was conducted in the absence of any commercial or financial relationships that could be construed as a potential conflict of interest.

## Footnotes

^1^SPSS includes a regression procedure called “two-stage least squares” which only implements the method described by Mandansky (1959) as “use of instrumental variables” to estimate the slope of the relation between *X* and *Y*. Use of this method requires extra variables with specific characteristics (variables which may simply not be available for the problem at hand) and differs meaningfully from the simpler and more generally applicable method to be discussed next
